# Lower Motor Neuron Findings after Upper Motor Neuron Injury: Insights from Postoperative Supplementary Motor Area Syndrome

**DOI:** 10.3389/fnhum.2013.00085

**Published:** 2013-03-18

**Authors:** Jeffrey E. Florman, Hugues Duffau, Anand I. Rughani

**Affiliations:** ^1^Neurosciences Institute, Maine Medical CenterPortland, ME, USA; ^2^Department of Neurosurgery, Gui de Chauliac Hospital, Montpellier University Medical CenterMontpellier, France; ^3^Division of Neurosurgery, Toronto Western Hospital, University of TorontoToronto, ON, Canada

**Keywords:** apraxia, hyporeflexia, motor cortex, spinal cord, supplementary motor area, spinal shock

## Abstract

Hypertonia and hyperreflexia are classically described responses to upper motor neuron injury. However, acute hypotonia and areflexia with motor deficit are hallmark findings after many central nervous system insults such as acute stroke and spinal shock. Historic theories to explain these contradictory findings have implicated a number of potential mechanisms mostly relying on the loss of descending corticospinal input as the underlying etiology. Unfortunately, these simple descriptions consistently fail to adequately explain the pathophysiology and connectivity leading to acute hyporeflexia and delayed hyperreflexia that result from such insult. This article highlights the common observation of acute hyporeflexia after central nervous system insults and explores the underlying anatomy and physiology. Further, evidence for the underlying connectivity is presented and implicates the dominant role of supraspinal inhibitory influence originating in the supplementary motor area descending through the corticospinal tracts. Unlike traditional explanations, this theory more adequately explains the findings of postoperative supplementary motor area syndrome in which hyporeflexia motor deficit is observed acutely in the face of intact primary motor cortex connections to the spinal cord. Further, the proposed connectivity can be generalized to help explain other insults including stroke, atonic seizures, and spinal shock.

Hyperreflexia and hypertonia are the classic upper motor neuron (UMN) signs thought to occur from the loss of corticospinal motor tract suppression of the spinal reflex arc. These “release signs” were described by Hughlings Jackson in 1931 as “positive signs” (Jackson et al., 1931), and have been suggested to be enhanced reflexes released by pyramidal lesions (Landau and Clare, 1959). However, hyporeflexia, atonia, and other lower motor neuron (LMN) signs are observed after acute central nervous system insults such as SMA syndrome and spinal shock. This observation may yield insight into functional connectivity underlying pathological spinal reflexes.

SMA syndrome has been described most commonly as a result of surgical resection of cortex anterior to the precentral gyrus (Laplane et al., 1977; Zentner et al., 1996). Classically, it follows a triphasic pattern, with an initial contralateral akinesia lasting several days that is often associated with preserved strength for involuntary movements. This is followed by a reduction in spontaneous activity of the contralateral limbs that lasts for days to weeks. If the dominant speech hemisphere is involved, then the early phase is also associated with expressive aphasia. It is worth emphasizing that the syndrome that follows resection of the SMA includes hemiparesis usually without hyperreflexia, and typically acute hyporeflexia is seen in this syndrome despite the unequivocal preservation of the primary motor cortex and its contributions to the corticospinal tract (Krainik et al., 2001).

Although the SMA has extensive projections through the motor systems, a key observation to derive an explanation for these classically LMN findings is that direct cortical stimulation of the primary motor cortex does not always cause motor movements immediately after the SMA syndrome occurs. This has been observed intra-operatively by the authors and described by others with motor evoked potentials following SMA resection (Zentner et al., 1996). Understanding this finding of blocked transmission from primary motor cortex neurons that end on alpha motor neurons of the spinal cord augments historic descriptions of the connectivity. With intact corticospinal tracts from the motor cortex, the lost response to cortical stimulation along with hyporeflexia appears consistent with more distal interruption, perhaps at the level of the spinal cord, caused by the loss of the SMA contribution to the corticospinal tract. This observation has been difficult to reconcile with conceptions of the anatomico-functional relationship between the SMA, primary motor cortex, and the spinal cord.

In this article, we review historic explanation for the acute hyporeflexia of UMN injuries and propose a theory implicating corticospinal tracts originating outside of the primary motor cortex. We hypothesize that the SMA contributions to the motor system provide a net inhibitory influence on the spinal cord and acute compromise is a dominant effector of acute hypotonia and hyporeflexia.

## Clinical Insights

Spinal shock offers the classically described paradigm of acute hypotonic plegia after CNS injury. Over the last two centuries, the teaching that has persisted is that this hyporeflexia is caused by loss of excitatory background descending input to the spinal motor neurons and interneurons leading to a hyperpolarization (Ashby et al., 1974; Ko et al., 1999; Ditunno et al., 2004). However, lost descending tonic influence is also used to explain the increased excitability that is associated with delayed spinal cord injury (Nielsen et al., 2007). Further, it is accepted that alpha motor neuron depression is not the sole source for reflex depression (Hiersemenzel et al., 2000). Other contributory metabolic, humoral, and structural mechanisms have been proposed to explain the temporal evolution of spinal shock to eventual hypertonicity and hyperreflexia, but they fail to clearly explain the initial reflex response and have clouded our interpretation of UMN function (Hiersemenzel et al., 2000; Ditunno et al., 2004). Even focal and incomplete injuries to the spinal cord, in anterior spinal artery infarct, for example, are similarly associated with an initial flaccid weakness (Suzuki et al., 1998; Millichap et al., 2007). In a complementary manner, the reflex recovery after supplementary motor cortex resection occurs over days to weeks as is expected after acute spinal cord injury (Dittuno and Ditunno, 2001; Krainik et al., 2001).

### Spinal reflex arc

**Figure 1:**
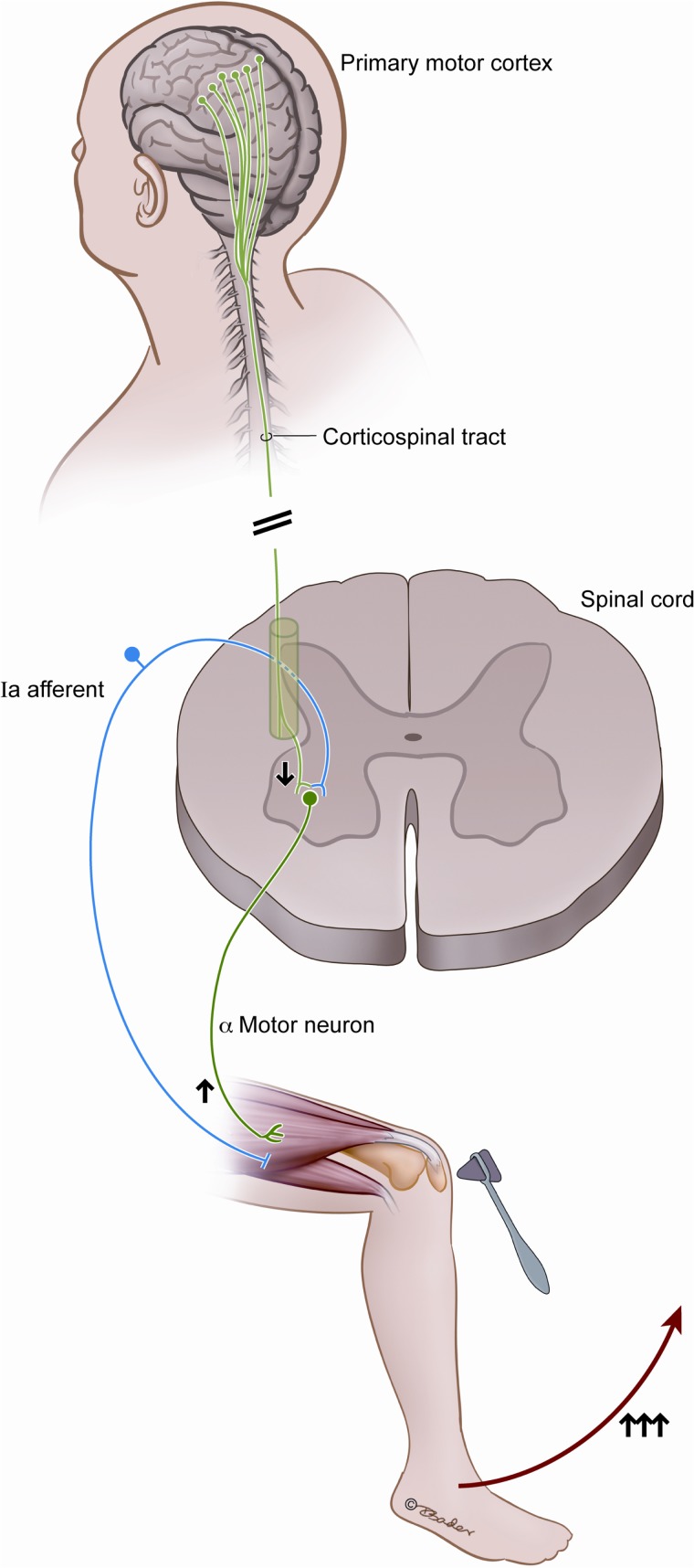
**This figure illustrates the classically described spinal reflex arc and the historically described contradictory changes in output resulting from corticospinal tract injury**. Arrows indicate the increased alpha motor neuron output as a result of injury to the corticospinal tract, despite the direct connection and stimulatory output from the motor cortex.

A reflex conveys an afferent stimulus to an effector via an integration center, and a simple physiologic version is the monosynaptic arc that underlies the deep tendon reflex (Figure 1). The responsiveness of the two neuron backbone is the result of an interplay between the local segmental inputs and descending influences. While the value of the tendon reflex has been appreciated since Erb in 1875 and serves as a “hard” sign in a clinical assessment, reflex responses can be variable and misleading (Louis and Kaufmann, 1996; Dick, 2003). The context of reflex responsiveness is often key to interpreting their significance, and the physiology at play is not always evident. UMN lesions are said to result in a net loss of inhibition that enhances tonic and phasic stretch reflex responses (Brashear and Elovic, 2010), however, this is not always clinically observed.

**Figure 2:**
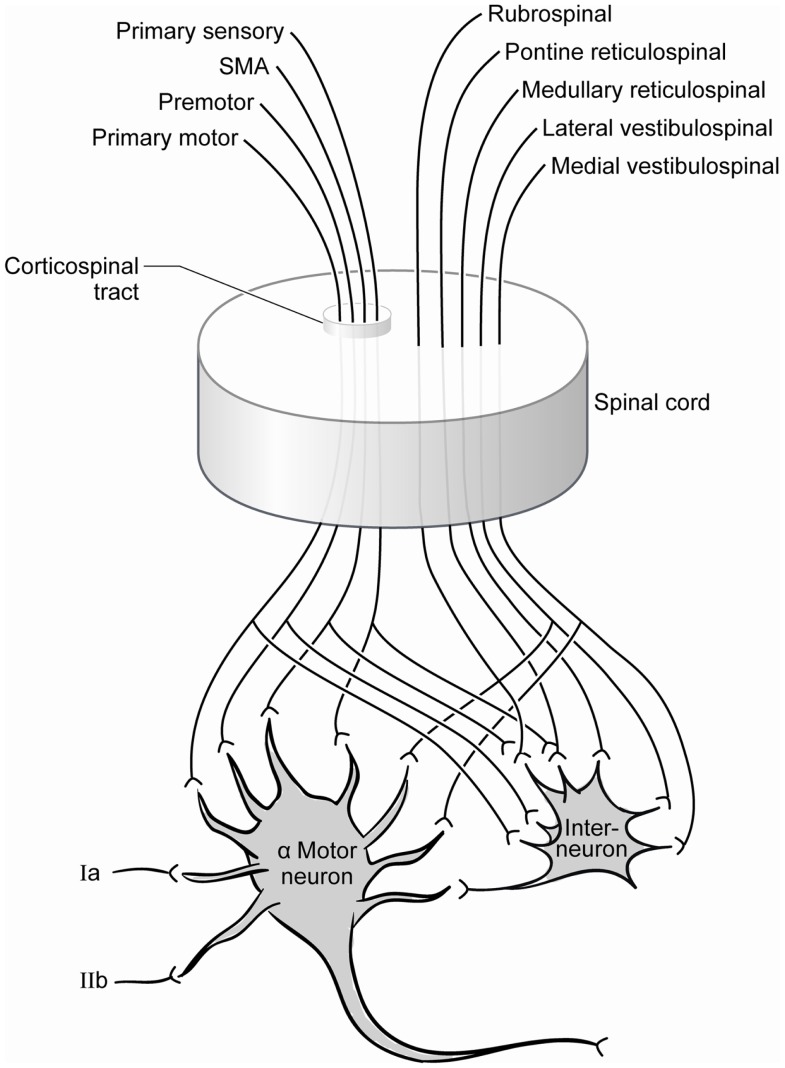
**Contemporary summary of the afferent pool of the alpha motor neuron**. Significant contributions include those from the spinal interneuron pool whose inputs include the same projections received from the corticospinal tract.

The extensive synaptic contributions to the monosynaptic tendon reflex complex are illustrated by exploring the afferent and efferent connectivity of the alpha motor neuron (Figure 2). Supraspinal input includes the corticospinal tract, the rubrospinal tract, the vestibulospinal tract; and segmental and intersegmental input includes the interneuron pool and extensive sensory afferents (Carpenter, 1991; Kingsley et al., 1996). There is a non-linearity to the input-output relationship of the motor neuronal pool, and the influence of some neuronal inputs may not be sufficient to independently achieve an excitation threshold but other neurons can facilitate (Emmanuel Pierrot-Deseilligny, 2012). Conversely, the postsynaptic output following simultaneous stimulation by two input neurons can be less than that following stimulation by only one of the neurons, a phenomenon called occlusion. As the interneurons play a large role in the ultimate response of the alpha motor neurons, afferent, and efferent contributions to this neuron pool may be consequential in the response to injury. While the nature of these interactions is not entirely understood for all contributors, it is clear that alpha motor neuron stimulation and suppression is achieved via a complex and poorly quantified afferent pool that influences the central state of the cells. The suprasegmental descending fiber systems normally maintain the spinal motor neurons in a state of readiness (Ropper et al., 2009), but the function of the cortex supplying the tract likely dictates the specific role.

### Cortical functions

An opportunity to study the corticospinal tracts is offered by the study of discrete surgical lesions, focal stroke, cortical electrical stimulation, EEG, and functional imaging. The differences in function and response to insult between areas supplying corticospinal neurons support the concept of alternate functions and mechanisms of action beyond pure motor activation. For example, the discrete functions of the precentral and postcentral gyri are relatively easily discernible with well-established principle roles of mediating primary motor and sensory functions, respectively (Penfield and Rasmussen, 1950; Uematsu et al., 1992; Salvan et al., 2006). Clinically illustrative is that pure sensory stroke is described after focal infarct to the postcentral gyrus (Derouesne et al., 1984; Kim, 1992), and resection of portions of the postcentral gyrus generally does not lead to motor deficit (Lewin and Phillips, 1952). Similarly, dedicated function of the primary motor cortex is supported by the dense and often irreversible focal motor deficit seen following even small focal insults (Pikula, 2011). Other areas contributing to the corticospinal tracts, such as the cingulate and premotor areas, may be integral to movement, but they do not have such evident “eloquent” function and their injury is not generally associated with permanent motor deficits (Lewin and Phillips, 1952). In short, except for the primary motor cortex, cortical areas contributing corticospinal tracts need not contribute essentially to motor neuron activation and are of ill-defined purpose in their capacity as efferents to the spinal cord.

Recognition of cortical inhibitory effects on motor function offers potential insights into connectivity that might participate in distal effects following injury. There is a sequential activation of “higher order motor areas” including the anterior cingulate, the SMA, and the inferior parietal lobe followed by activation of “executive areas” including the posterior portion of the SMA and the primary motor area (Ball et al., 1999). Evidence strongly supports an inhibitory function to the SMA with reciprocal activation of this area and the primary motor area. Ball et al. (1999) suggest that motor initiation involves a release of inhibition of the executive areas by the intermediate SMA, and this is also supported by non-human primate studies (Richter et al., 1997). This concept of negative motor areas and inhibitory effects is well described (Luders et al., 1995) and the SMA is consistently identified as playing this inhibitory role in voluntary movement. The SMA, pre-SMA, inferior frontal gyrus, and the medial frontal gyrus have been heavily implicated in the planning of actions and in the ability to stop an action in progress (Sharp et al., 2010; Swann et al., 2012). Functional imaging has further elucidated the relationship between regions recruited to achieve inhibitory control with premotor areas being integral (Swann et al., 2012).

Brain death and some focal seizure syndromes may hint at further clinical insight into hypotonia and diminished reflexes when the primary physiology involves the CNS. While brain death can often be attributed to an isolated brain injury, the associated plegia is confidently attributed to supraspinal compromise but not to a more focal insult. Atonic seizures have been localized to negative motor areas anterior to the supplementary motor area (Luders et al., 1995). “Negative” motor areas are hypothesized by Luders and colleagues to play an important role in suppressing movement during the planning of an action or actions. Electroencephalographic data derived from patients and non-human primates corroborates this. For example, the origin of ictal onset sudden atonic collapse of the legs, known as “drop attacks,” may be the supplementary motor area itself (Meletti et al., 2000; Satow et al., 2002; Saeki et al., 2009). Further yet, there is evidence that negative myoclonus with focal epilepsy may be related to a decrease in the excitatory input on spinal motor neurons through direct corticospinal connection (Luders et al., 1995). Insights into complex supratentorial events such as these support the theme of UMN functionality as a significant participator in hyporeflexia motor deficits.

### Descending supraspinal inputs

Conventional teaching holds that only one-third of the corticospinal tract arises from the primary motor cortex and one-third from the supplementary motor cortex and one-third from the primary sensory cortex (Carpenter, 1991). Studies also suggest origins from the cingulate gyrus in primates (Luppino et al., 1994). Interestingly, diffusion tensor imaging suggests individual variation and increasing diversity of cortical contributions to the corticospinal tract with increasing age (Kumar et al., 2009). Assumptions about the somatotopy and function of the corticospinal tracts have been revisited over time. Many fibers of the corticospinal tract share similar projections (Dum and Strick, 1996) and one of the dominant functions is a corticobrachial outflow tract (Levi et al., 1996). However, many observations challenge the idea that the role of this entire descending pathway is to serve direct stimulatory motor control. For example, the primary motor cortex has monosynaptic projection to alpha motor neurons, propriospinal neurons, and segmental interneurons (Emmanuel Pierrot-Deseilligny, 2012); and the descending tracts communicate with multiple interneurons, travel to the ipsilateral and contralateral spinal cord, and branch in varying degrees (Chiappa et al., 1991). Subsequently, the corticospinal tract is perhaps best thought of as comprised of multiple subsystems involved in various aspects of motor control (Armand, 1984).

Extrapyramidal tracts also serve motor function and can effect reflex function but primarily act indirectly on the alpha motor neurons. Aside from the corticospinal inputs, descending tracts originate in numerous sites mostly within the brainstem and they modulate movements and participate in tone along with tracts originating in the cerebellum (Carpenter, 1991) as schematically illustrated in Figure 2. Since Sherrington’s century old descriptions, decerebrate rigidity has been associated with transection of the midbrain between the colliculi, and hyporeflexia is expected with transection below (Sherrington, 1898, 1910). Over time, however, some of Sherrington’s physiologic explanations have fallen short and there is increasing recognition of the complexities of the neural systems involved (MacKay-Lyons, 2002; Bouyer and Rossignol, 2003a,b; Matthews, 2004; Stuart, 2005). While animal models of decerebrate rigidity have proven some utility in understanding the role of descending influences on posture, Sherrington’s intensive study left him with the conclusion that the pyramidal tracts could not be implicated in decerebrate rigidity as a cause or remedy (Davis and Davis, 1981).

### Interneuronal connectivity

The spinal cord serves not only as a conduit for bidirectional information flow between the brain and periphery, but also harbors circuitry that is believed to independently subserve some motor functions such as for locomotion (Iglesias et al., 2008). Multiple central and peripheral inputs provide influence on the spinal neurons, and the cerebral and cerebellar cortices directly and indirectly connect with the LMNs (Jurgens, 1984). The variety of synaptic influences on the motor neurons are illustrated by the array of synaptic locations – be it axosomatic, axodendritic, and axoaxonal (Moriizumi et al., 1989; Motorina, 1991). While the neurotransmitters of the corticospinal tract are the excitatory transmitters glutamate and aspartate, it is the targets of those neurons that determine the ultimate effects (Giuffrida and Rustioni, 1989; Valtschanoff et al., 1993). The ultimate integration of descending motor signals, the local response to afferent spinal cord input, and communication back to the cortex is mediated at the level of the spinal neurons including interneurons.

Even with evidence of similar projections from the varied cortical motor areas to the spinal cord (Dum and Strick, 1996), the laminar sites of synapse vary substantially, at least in quantity, between the SMA and primary motor area (Maier et al., 2002). The different spinal laminar sites of termination of the crossed and uncrossed corticospinal tracts within the spinal cord furthers the concepts of physiologic and functional segregation between these tracts (Carpenter, 1991). More evidence of contrast is seen in electrophysiologic study of the SMA and primary motor cortex with apparent differential contributions to motor control (Maier et al., 2002). While even non-synaptic presynaptic modulation of neurotransmitter release has been suggested (Rudomin and Schmidt, 1999), the modulation of excitability of the intrinsic spinal circuitry is likely mediated via spinal interneurons and this may enable them to serve to modulate the different corticomotor inputs (Prut and Fetz, 1999; Bizzi et al., 2000; Maier et al., 2002).

The complexity of the contribution of interneurons to the motor system for locomotion, postural maintenance, and reflex responses is evidenced throughout the literature with acknowledgment that our understanding is primarily derived from limited animal studies. Even those interneurons that participate in locomotion as “last order” interneurons have pyramidal and extrapyramidal descending inputs influence their actions and these inputs can be excitatory and/or inhibitory. Further, the stimuli provided by interneurons to the motor neurons can be excitatory or inhibitory, non-reciprocal or reciprocal with movements (Nielsen et al., 2007), and are of uncertain priority for given tasks (Brownstone and Bui, 2010). In humans, there remains ambiguity about which interneurons are involved and their precise connectivity to support postural, reflex, and voluntary movement, but there is likely strong corticospinal excitation of interneurons inhibiting premotor neurons (Marchand-Pauvert et al., 1999). While there are many classes of interneurons, inhibitory modulation of transmitter release involved in presynaptic control can involve activation of GABAergic (Rudomin and Schmidt, 1999) or glycinergic interneurons (Brownstone and Bui, 2010). Such architecture has been described with contributions to the inhibitory interneurons and premotor neurons from afferent inputs including the group I and II afferents (Marchand-Pauvert et al., 1999; Iglesias et al., 2008).

Pyramidal tract projection to spinal interneurons has been known about for more than three decades and the ability of this architecture to manipulate reflexes is known (Rothwell, 1987). As to the precise interaction that explain reflex responses to corticospinal injury, stretch reflexes may involve polysynaptic pathways which are subject to the influence of a variety of descending and segmental inputs (Rothwell, 1987). Even cortical communication appears influential in the neuronal responsiveness. Described more than a century ago by Jendrassik, potentiation of stretch reflexes by contraction of remote muscles is now felt to be related to decreased excitability of inhibitory intracortical pathways participating in corticospinal communication (Tazoe et al., 2007). In addition to such evidence for cortical participation in reflexes, modulation can occur through many phases of movement planning and execution (Prut and Fetz, 1999).

Supraspinal control of interneurons has been described in human electrophysiological study with connection likely between the corticospinal tracts and non-reciprocal, group Ib, interneurons (Marchand-Pauvert et al., 1999; Iglesias et al., 2008). Also, cortical inhibition is at least in part likely mediated by direct monosynaptic corticospinal projections to group Ia inhibitory interneurons (Jankowska et al., 1976), demonstrating the potential for stimulatory and inhibitory influence of the corticospinal tracts. Lost inhibitory input from descending tracts has previously been posited as a contributor to depressed reflexes after spinal cord injury (Chen et al., 2001), and inhibition of interneurons may contribute (Lundberg, 1979; Lavrov et al., 2006).

## Toward a Unified Theory

This article builds upon the historical expectation of classic UMN signs, such as hypereflexia, when they are often not the acute response seen after CNS injury. Immediate hyporeflexia is a hallmark and consistent finding after many acute brain and spinal cord insults. Despite the failings of the historic explanations, extant descriptions of CNS connectivity and function support the concept of an inhibitory role for portions of the cortex, descending tracts, and spinal neurons; and a putative connectivity involving the related architecture can help explain hyporeflexia as a true acute UMN finding. The classic but tardy UMN findings that occur after CNS injury have been attributed to many mechanisms localized to the spinal cord mostly at the level of the interneurons (Ditunno et al., 2004).

**Figure 3:**
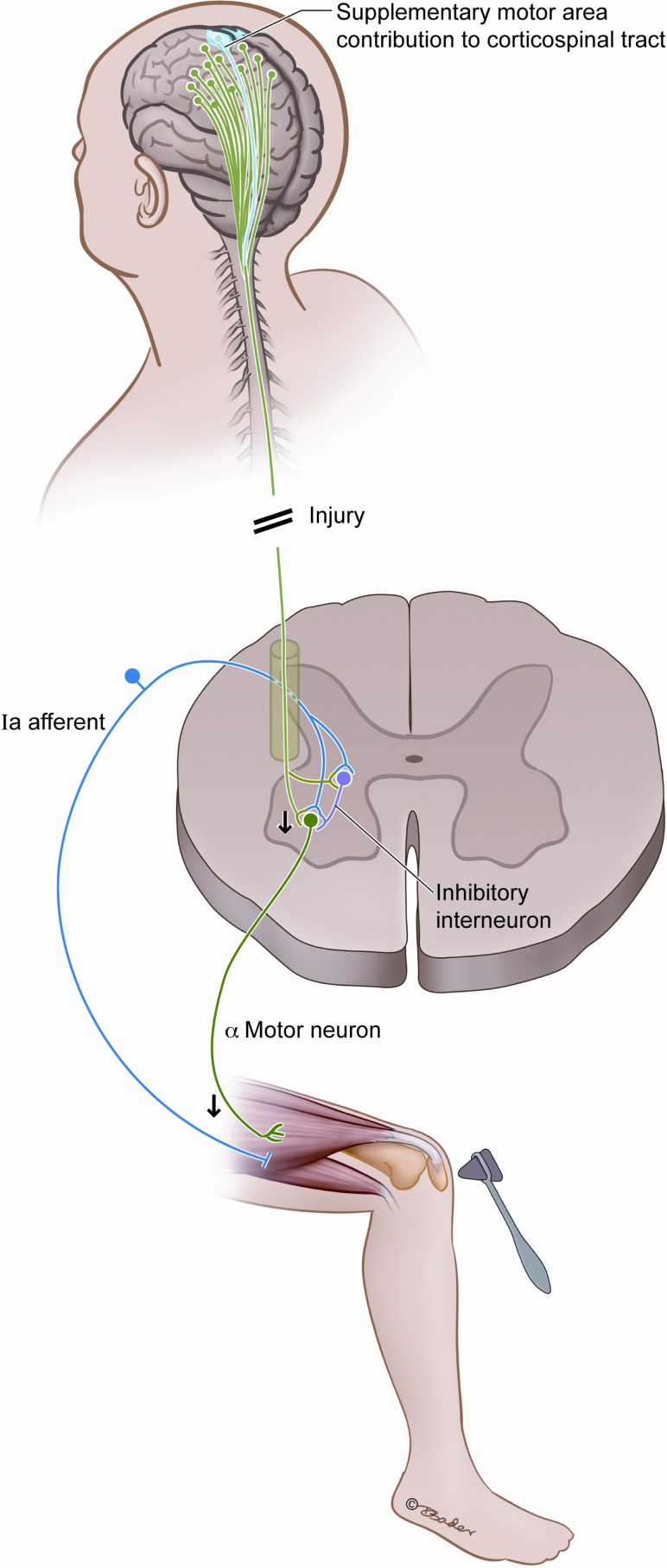
**Postulated interplay between the SMA and the spinal reflex arc**. We hypothesize that compromise of the SMA fibers diminishes reflex responsiveness by decreasing the excitability of alpha motor neurons, and this is a source of acute hyporeflexia after upper motor neuron injury such as spinal shock and SMA syndrome.

The theory we propose revolves around corticospinal fiber tracts originating outside of the primary motor cortex, in particular those from the SMA. This is in keeping with a central role of the SMA as an inhibitor of motor movement and consistent with the clinical and electrophysiologic responses seen after SMA resection (Schucht et al., 2012). While the descending tracts are stimulatory in their neurotransmitters, the recipient neurons act to suppress and inhibit movement in the spinal cord as a powerful motor modulator that engages postural, voluntary, and reflex functions. As illustrated in Figure 3, there are competing SMA signals to inhibitory spinal interneurons and to the alpha motor neurons that these interneurons stimulate. In the normal state, these signals likely modulate and coordinate planned actions by balancing the direct excitation of motor neurons and the indirect inhibition via the inhibitory spinal interneurons. Corticospinal fibers not arising from the primary motor cortex can increase the excitability of motor neurons (Lemon, 2008), and their compromise would be expected to decrease the excitability of the motor neurons. We propose that injury to the SMA results in a net loss of excitation to the alpha motor neuron, via decreased inhibition of inhibitory interneurons, and this underlies acute hyporeflexia.

The postulated connectivity to explain the reflex responses to CNS injury is notably consistent with that described for sympathetic activity after acute spinal transection. It has been proposed that spinal transection abolishes descending excitation of sympathetic neurons either via direct connection to the sympathetic preganglionic neurons or indirectly via interneurons; and transection also compromises descending inhibition of spinal systems with excitatory input to sympathetic preganglionic neurons (Schramm, 2006). It is this type of multidimensional interactions of supraspinal influence on spinal neurons that likely underlies the reflex responses after CNS injury.

The remarkable complexity of the pathophysiology that underlies clinical responses to insult are irresolvable with contemporary knowledge, and certainly there are often multiple pathways participating in a dynamic manner. Even in trying to strongly implicate portions of the corticospinal tract in hyporeflexia after injury, such postulations may prove inadequate given the numerous potential influences on the gain of the monosynaptic tendon reflex. While widespread projection from the SMA to the intermediate zone of the spinal cord suggests significant involvement of interneurons (Maier et al., 2002), direct connection between the SMA and the alpha motor neurons could also be a significant participant in effecting the clinical realities of acute CNS injury. Cortico-motoneuronal connections originating from the SMA and the primary motor cortex converging on single motor neurons is evidenced (Maier et al., 2002). Other corticospinal communication, via the primary sensory cortex for example, might also participate but isolating the SMA and its inhibitory functional connectivity supports at least one cohesive and consistent mechanism for hyporeflexic and hypotonic acute paresis. Isolating robust anatomical and physiological support is challenging given the range of temporal and spatial resolution required to provide supporting evidence. Corroborating evidence could perhaps emerge from combining functional disruption studies, using modalities such as transcranial magnetic stimulation, in tandem with *in vivo* electrophysiological recording.

Hurdles to understanding human reflex responses to injury include the breadth of clinical scenarios, dramatic temporal evolution of responses over time following insults, the difficulties correlating animal studies to humans, the functional heterogeneity of areas such as the SMA, the potential for individual variation in movement related cortical responses, the rarity of small focal lesions to the related areas, the limited resolution of EEG and imaging, the limited spatial resolution of cortical and subcortical stimulation, the difficulties performing the human electrophysiological studies that would be most valuable, and the remarkable complexity of the connectome (Ball et al., 1999; Chung et al., 2005; Sumner et al., 2007). The study of neurologic events that are fascinating and sometimes counterintuitive will certainly stimulate additional exploration and challenge historical assumptions. At a minimum, the hypothesis put forward here should challenge the notion that hyporeflexia is simply a LMN finding as it is the logical and expected response to many UMN injuries. The authors favor the theory that SMA contributions to the corticospinal tract and in turn to alpha motor neurons and spinal interneurons underlie clinical observations of many such insults seen by neurosurgeons.

## Conflict of Interest Statement

The authors declare that the research was conducted in the absence of any commercial or financial relationships that could be construed as a potential conflict of interest.
